# How Rice Plants Response to Abiotic Stresses

**DOI:** 10.3390/ijms241612806

**Published:** 2023-08-15

**Authors:** Baohua Feng, Jie Xiong, Longxing Tao

**Affiliations:** 1 National Key Laboratory of Rice Biology, China National Rice Research Institute, Hangzhou 310006, China; fengbaohua@caas.cn; 2 College of Life Sciences and Medicine, Zhejiang Sci-Tech University, Hangzhou 310018, China; jiexiong@zju.edu.cn

With the combustion of fossil fuels, unequal and unsustainable energy and land use, and irrational human activities, greenhouse gas emissions remain high, which leads to global warming. The IPCC’s Sixth Assessment Report shows that from 2011 to 2020, the surface temperature increased by 1.09 °C compared to the period of 1850–1900, and the global average temperature is projected to increase by 1.5 °C or more in the next 20 years. Global warming results in more and more frequent and intense extreme weather events. Extreme weather events such as droughts, floods, and heatwaves can lead to reduced crop yields, lower quality, and unstable food supply, thereby affecting global food security and causing significant economic and environmental impacts on human society.

Rice (*Oryza Sativa* L.) is one of the most important food crops, feeding over 3 billion people worldwide. Rice provides 21% of the energy and 15% of the protein needed for humans. Besides its nutritional and economical importance, rice also serves as a model system to monocots in plant biology research and is the first crop plant to have its genome fully mapped. Given the preponderant role of rice as a major food resource, rice’s physiology and regulation has become a top focus of the crop science community.

Rice is a fast-growing crop that requires a large amount of water, has strong photosynthetic activity, and is sensitive to temperature. It has high fertilizer requirements and the ability to be densely planted. Unsuitable abiotic stressors can affect the growth, development, and yield quality of rice, posing a threat to its safe production. For example, drought conditions can hinder plant growth and reduce yield. Additionally, drought can lead to a decrease in the nutritional value of rice grains, such as reduced starch content and inhibited protein synthesis. High salt concentrations can affect the ability of rice roots to absorb water and nutrients, resulting in poor plant development and reduced yield. Moreover, ion stress in saline–alkali environments can disrupt the metabolic processes of rice and affect grain quality by, for example, reducing starch content and increasing impurity levels. High temperatures can inhibit rice photosynthesis and respiration, leading to poor plant growth and reduced grain yield. High temperatures can also affect rice starch synthesis as well as the synthesis of proteins and nutrients, resulting in a decline in rice quality. Low temperatures can delay rice growth, prolong the reproductive period, and reduce yield. Low temperatures can also affect rice starch synthesis and fatty acid metabolism, impacting grain quality. Although there have been numerous studies on the response and regulatory mechanisms of rice to abiotic stressors in recent years, our understanding is still limited due to the complex nature of rice. Thus, we organized a Special Issue titled “How Rice Plants Response to Abiotic Stresses” of the International Journal of Molecular Sciences. This issue includes nine contributions which provides new information on the mechanism of rice plants’ responses to abiotic stresses.

Chen et al. [1] identified a highly salt-sensitive mutant ss3 from the mutant library of Nipponbare, and a map-based cloning approach was used to isolate the gene SS3, which encodes mannose-1-phosphate guanylyltransferase. The author found that ss3 mutants decreased the ascorbic acid (AsA) content and increased reactive oxygen species (ROS) levels compared with the wild type (WT) under salt treatment. Also, exogenous AsA as able to restore the salt tolerance of the ss3 mutant, indicating that inhibition of AsA synthesis was an important factor in the salt sensitivity of the mutant.

Zhu et al. [2] used two rice genotypes, Zhefu802 (heat-resistant) and its near-isogenic line (*Fgl*, heat-sensitive), to study the effect of ABA on the heat response of rice at the flowering stage. The results showed that exogenous ABA significantly increased the seed-setting rate of rice under heat stress. Similarly, exogenous ABA increased the trehalose content, key enzyme activities of trehalose metabolism, ATP content, and F1Fo-ATPase activity. Importantly, the opposite results were observed in plants treated with Flu. Thus, the author deduced that ABA could improve rice thermo-tolerance by affecting trehalose metabolism and ATP consumption.

The overexpression of *OsABT* could enhance salt tolerance in rice seedlings by regulating root activity, relative conductivity, malondialdehyde and H_2_O_2_ content, and O_2_^−^ production rate [3]. In the study, the author found that lower Na+ and higher K+/Na+ ratios, as well as higher expression of salt-related genes *OsSOS1* and *OsHAK5*, were observed in the *OsABT* overexpression lines. Thus, the author thought that the *OsABT* could improve the salt tolerance in rice seedlings by inhibiting reactive oxygen species accumulation, thereby regulating the intracellular Na+/K+ balance, ABA content, and ABA signaling pathway.

Wang et al. [4] investigated dry matter accumulation and translocation, grain filling, and enzyme activities involved in the starch synthesis of superior and inferior grains of rice from 2016 to 2017. The author found that dry matter accumulation was significantly reduced in organs under drought stress at the jointing-booting stage and eventually decreased the grain number per panicle and the thousand-grain weight. The high content of NSCs in the stems and sheaths plays a key role in resisting drought stress. The grain-filling rate decreased significantly with the increase in the drought stress. The author also found that drought stress changed the starch synthesis strategy of superior and inferior grains. The amylose content was decreased and amylopectin synthesis was enhanced under drought stress, especially in inferior grains.

Zhang et al. [5] studied the mechanism of higher saline–alkali tolerance in autotetraploid rice 93-11T compared to the diploid rice 93-11D through physiological, hormonal, and transcriptomic measurements. The results showed that 93-11T exhibited a higher osmotic regulation based on the regulation of cuticular wax synthesis; higher antioxidant capacity, including SOD, POD, and lignin biosynthesis; and higher SA levels despite lower IAA levels than 93-11D under saline–alkali stress conditions.

Kaur et al. [6] conducted physiological, biochemical, and molecular analyses of two rice cultivars, one drought-tolerant (N 22) and one drought-sensitive (IR 64), under drought stress. The results suggested that stress signaling, redox homeostasis, antioxidant activity, biosynthesis of secondary metabolites, linoleic acid metabolism, ABA biosynthesis, and transcription factors are mainly responsible for the observed reproductive-stage drought stress tolerance in N 22.

Luan et al. [7] found that nine 1,3-β-glucanases (BGs) belonging to the carbohydrate metabolic pathway were expressed differently during heat stress through RNA-seq analysis. Then, the author identified the BGs and glucan-synthase-likes (GSLs) in rice and processed the analyses of gene gain and loss, phylogenetic relationships, duplication, and syntenic relationships. They deduced that there might be an environmental adaption based on BGs and GSLs during evolution through the submicrostructure and dry matter distribution analysis results. However, the exact underlying mechanism needs more investigation.

Qiu et al. [8] integrated the transcriptomic and epigenetic sequencing data analyses to identify the key TFs-*OsbZIP14* in rice in response to heat stress. The author found that *OsbZIP14* could positively contribute to heat tolerance through the concerted action of *OsbZIP58* and *OsbZIP14*, while dysfunction of *OsbZIP14* in WT resulted in higher heat stress susceptibility, associated with cell membrane damage and H_2_O_2_-induced cell death.

Dynamic transcriptomic analyses of the two cultivars (T11: heat-tolerant and T15: heat-sensitive) were conducted after 0 min, 10 min, 30 min, 1 h, 4 h, and 10 h of 42 °C heat stress [9]. The author found that several pathways rapidly responded to heat stress, such as protein processing in the endoplasmic reticulum, glycerophospholipid metabolism, and plant hormone signal transduction. They also found that the tolerant cultivar responded more rapidly and intensively to heat stress compared to the sensitive cultivar. Furthermore, the author identified 27 candidate genes by combining data from a GWAS and RNA-seq analysis.

The traditional physiological parameters, including ROS balance, antioxidant capacity, and Hsps, played an important role in studying abiotic stress responses in the past, while sugar signaling and energy metabolism are becoming more and more important in the study of abiotic stress. Studies have demonstrated these abiotic stress parameters involved in heat, drought, salt, and alkali conditions [2,4,5,6]. Referring to the molecular level, Chen et al. [1] and Wen et al. [3] demonstrated some genes involved in salt and alkali responses in rice. In fact, plants’ stress responses are definitely not controlled by single gene, so it is important to study the physiological metabolic pathways and processes. Luan et al. [7], Qiu et al. [8], and Cai et al. [9] studied heat response mechanisms such as the carbohydrate metabolic pathway, glycerophospholipid metabolism, and plant hormone signal transduction through transcriptomic data. In conclusion, the three aspects of physiological, single-gene regulator, and omics analyses play important roles in the study of rice responses to abiotic stress. This Special Issue provides valuable information on rice responses to abiotic stresses, and lays a foundation for the study of abiotic stress in rice breeding.
